# Language and Cognitive Performance in Children with Well-Controlled Asthma: Associations with Serum pNF-H and Neurogranin

**DOI:** 10.3390/children13101393

**Published:** 2026-10-13

**Authors:** Merve Savaş, Senanur Kahraman Beğen, Ece Akbayır, Esra Yücel, Ayça Kıykım, Fatih Dilek, Erdem Tüzün, Arzu Temizyürek, Seyma Dumur, Yusuf Elgormus, Hafize Uzun

**Affiliations:** 1 Department of Speech and Language Therapy, Faculty of Health Sciences, Istanbul Atlas University, Istanbul 34403, Türkiye; merve.savas@atlas.edu.tr (M.S.); senanur.kahraman@atlas.edu.tr (S.K.B.); ece.akbayir@atlas.edu.tr (E.A.); 2 Department of Pediatric Allergy and Immunology, Cerrahpaşa Faculty of Medicine, Istanbul University-Cerrahpaşa, Istanbul 34098, Türkiye; esra.yucel@iuc.edu.tr (E.Y.); ayca.kiykim@iuc.edu.tr (A.K.); 3 Department of Pediatrics, Faculty of Medicine, Istanbul Atlas University, Istanbul 34403, Türkiye; drfatihdilek@yahoo.com (F.D.); yusuf.elgormus@atlas.edu.tr (Y.E.); 4 Department of Neuroscience, Aziz Sancar Institute of Experimental Medicine, Istanbul University, Istanbul 34452, Türkiye; erdemtuzun@istanbul.edu.tr; 5 Neurovascular Research Unit, Department of Radiology, Massachusetts General Hospital, Harvard University, Boston, MA 02114, USA; atemizyurek@mgh.harvard.edu; 6 Department of Medical Biochemistry, Faculty of Medicine, Istanbul Atlas University, Istanbul 34408, Türkiye; seyma.dumur@atlas.edu.tr

**Keywords:** childhood asthma, cognitive function, language development, neuroinflammation, neurogranin, pNF-H

## Abstract

**Highlights:**

**What are the main findings?**

- Children with asthma showed differences in full-scale IQ and verbal and attention/working-memory-related indices compared with healthy controls.
- Serum pNF-H levels showed the clearest difference between children with asthma and healthy controls, and serum neurogranin levels were linked to specific measures of cognition and language.

**What are the implications of the main findings?**

- Even in children whose asthma is well-controlled, subtle differences may be observed in certain cognitive and language domains.
- Biomarkers related to neuroaxonal and synaptic processes may be associated with these subtle cognitive and language differences in well-controlled childhood asthma.

**Abstract:**

**Background/Objectives:** Childhood asthma is increasingly viewed as a systemic condition that may also affect neurodevelopmental processes. We examined language and cognitive performance and their associations with serum biomarkers of neuroinflammation, neuroaxonal integrity and synaptic function in school-aged children with clinically well-controlled asthma. **Methods:** In this cross-sectional study, 67 children (33 with asthma and 34 healthy controls) underwent the Wechsler Intelligence Scale for Children–Revised (WISC-R), the Turkish Nonword Repetition Test (TAST), the Turkish School-Age Language Development Test (TODİL), sleep and psychosocial scales, and audiometric screening. Serum interleukin-4 (IL-4), IL-13, C-X-C motif chemokine ligand 13 (CXCL13), brain-derived neurotrophic factor (BDNF), nerve growth factor (NGF), chitinase-3-like protein-1 (YKL-40/CHI3L1), triggering receptor expressed on myeloid cells 2 (TREM2), monocyte chemoattractant protein-1 (MCP-1), synaptosomal-associated protein 25 (SNAP-25), neurogranin, and phosphorylated neurofilament heavy chain (pNF-H) were measured using enzyme-linked immunosorbent assay (ELISA). Groups were compared with Mann–Whitney U and chi-square/Fisher tests and with age- and sex-adjusted regression using HC3 robust standard errors, with Benjamini–Hochberg (BH) false-discovery-rate (FDR) correction within predefined outcome families; within the asthma group, biomarkers were related to language and cognitive outcomes in exploratory multiple regression. **Results:** Serum pNF-H was higher in the asthma group (*p* = 0.001; pFDR = 0.012), remained higher after age and sex adjustment (*p* = 0.002), and was robust to outlier handling. Neurogranin was higher in the unadjusted comparison (*p* = 0.012), marginal after panel-level FDR (pFDR = 0.065), and significant after adjustment (*p* = 0.003). Several WISC-R indices, most clearly full-scale IQ, were lower in the asthma group, favoring controls. Within the asthma group, neurogranin was positively associated with WISC-R vocabulary and picture completion and TODIL organizing. **Conclusions:** Even in well-controlled asthma, specific language and cognitive domains may differ from those of healthy peers, alongside higher peripheral biomarkers related to neuroaxonal and synaptic processes. These findings are exploratory and require confirmation in larger, longitudinal samples.

## 1. Introduction

Asthma is a common chronic inflammatory airway disease of childhood characterized by reversible airflow obstruction and type-2 inflammation involving interleukin (IL)-4 and IL-13 [1,2]. Beyond its respiratory manifestations, asthma is increasingly recognized as a systemic condition that may affect sleep, quality of life, academic functioning, and neurodevelopment. Allergic diseases, including atopic dermatitis, allergic rhinitis, and asthma, often begin in early childhood and impose a substantial burden on affected children and their families [3].

Allergic inflammation may also influence central nervous system function. The activation of basophils, mast cells, and eosinophils releases proinflammatory mediators [4], while circulating cytokines and peripheral immune cells may affect blood–brain barrier permeability and contribute to neuropsychiatric processes [5,6]. The increased coexistence of allergic and neurodevelopmental or psychiatric conditions further suggests shared underlying mechanisms [7]. In children with asthma, disease severity has been associated with cognitive, behavioral, social, and academic difficulties [8,9], involving domains such as attention, processing speed, executive function, language, learning, memory, and visuospatial ability [10]. A recent systematic review and meta-analysis of 104,975 participants found greater attention difficulties in children with asthma than in healthy controls, whereas memory performance did not differ significantly, suggesting that asthma-associated cognitive effects may be domain-specific [11].

Despite growing evidence regarding cognition and academic performance, the linguistic subskills potentially affected in childhood asthma remain insufficiently characterized. Language is a central developmental domain influencing communication, psychosocial adjustment, and academic progress [12,13]. Its assessment may combine norm-referenced and descriptive approaches, which provide complementary information [14]. Interpretation is complicated by common comorbidities: otitis media with effusion may impair hearing and cognitive development [15]; allergic rhinitis is associated with sleep disturbance, fatigue, poor concentration, and cognitive difficulties [16]; sleep disorders may themselves affect cognition and mental health [17]; and anxiety or other psychiatric comorbidities can further influence performance [18]. These factors were therefore assessed, including audiological screening with tympanometry and pure-tone audiometry.

Several mechanisms may link asthma to central nervous system function. Chronic systemic inflammation and circulating cytokines may alter neural signaling [5], while intermittent hypoxia and fragmented sleep may adversely affect attention and memory [17,19]. Neuroimaging and cerebrospinal-fluid studies in adults have also suggested associations between asthma and alterations in brain microstructure, neuroinflammatory activity, synaptic integrity, and cognitive function [20,21].

Within the broader biomarker panel, phosphorylated neurofilament heavy chain (pNF-H) and neurogranin were included as complementary indicators of neuroaxonal integrity and synaptic function rather than as direct markers of neuroinflammation. pNF-H is a phosphorylated component of the axonal cytoskeleton that may enter biological fluids following the disruption of neuroaxonal integrity, whereas neurogranin is a postsynaptic protein involved in synaptic plasticity, learning, and memory [22,23,24]. These markers were considered biologically relevant because systemic inflammation, intermittent hypoxia, and sleep disruption associated with asthma may influence neuroaxonal and synaptic processes during childhood [17,19,20]. However, evidence relating these peripheral biomarkers to language and cognition in pediatric asthma remains limited; their evaluation in the present study was therefore exploratory and hypothesis-generating.

We evaluated cognitive, language, sleep, and psychosocial functioning together with a multi-analyte serum biomarker panel in school-aged children with clinically well-controlled asthma. We examined between-group biomarker differences and explored associations between selected biomarkers and language–cognitive outcomes within the asthma group. Because the study was not powered a priori, all findings were interpreted as preliminary and hypothesis-generating.

## 2. Methods

### 2.1. Design and Ethics

This cross-sectional observational study was conducted at İstanbul Atlas University Hospital between April 2023 and April 2026 in accordance with the principles of the Declaration of Helsinki. The study protocol was approved by the İstanbul Atlas University Non-Interventional Scientific Research Ethics Committee (approval date: 26 October 2022; approval no. E-22686390-050.99-21202). Written informed consent was obtained from the parents or legal guardians of all participants, and age-appropriate assent was obtained from the children. No a priori sample size calculation was performed because the study was designed as an exploratory investigation.

### 2.2. Participants and Eligibility

Sixty-seven children were enrolled (33 asthma, 34 healthy controls). Asthma was confirmed by a pediatric allergy/immunology specialist using clinical assessment and pulmonary function testing, and all participating children had clinically stable, well-controlled disease. Children with asthma remained under routine follow-up in the Pediatric Allergy and Immunology clinic; however, the present analysis was based on a single assessment point and did not include longitudinal reassessment of cognitive, language, or biomarker outcomes. Controls had no chronic disease, diagnosed hearing loss or neuropsychiatric history. Exclusion criteria included comorbid chronic inflammatory disease, immunodeficiency, diabetes, chronic kidney disease, malignancy, rheumatological disease, malnutrition, diagnosed hearing loss, known neuropsychiatric disorder, non-attendance at school, or a Wechsler Intelligence Scale for Children–Revised (WISC-R) full-scale score below 70. The observed age range was approximately 7–17 years.

### 2.3. Demographic and Clinical Data

Age, sex, anthropometry and education were recorded for all participants; for the asthma group, age at onset, disease duration, severity, control level, comorbid atopic conditions, medication use and follow-up information were documented, with severity and control assessed by the specialist physician.

### 2.4. Cognitive and Language Assessment

Cognitive ability was assessed with the WISC-R, yielding verbal, performance and full-scale IQ, factor indices and subtest scores; full-scale IQ was the principal cognitive outcome. Language was assessed with standardized tests (the TAST and TODİL); because TODİL yields ordinal normative levels, it was analyzed and reported by category/level rather than by mean score. Cognitive ability was assessed using the WISC-R, yielding verbal, performance, and full-scale intelligence quotient (IQ) scores, factor indices, and subtest scores; full-scale IQ was the principal cognitive outcome. Language was assessed using the Turkish Nonword Repetition Test (TAST) and the Turkish School-Age Language Development Test (TODİL). Because TODİL yields ordinal normative levels, it was analyzed and reported by category/level rather than by mean score. The WISC-R intelligence quotient and index scores were age-standardized according to the test’s normative procedures. TODİL outcomes were interpreted using age-based normative levels, whereas the TAST total-correct score was not age-standardized.

### 2.5. Sleep, Psychosocial and Audiological Measures

Sleep was assessed with the Sleep Disturbance Scale for Children and psychosocial functioning with parent-report scales (behavioral competence/problem and a child anxiety scale). Because otitis media with effusion is a frequent, easily missed comorbidity in asthma, middle-ear pressure was assessed by tympanometry and hearing thresholds by pure-tone audiometry; children with a pure-tone average > 20 dB HL were excluded from the relevant analyses.

### 2.6. Serum Biomarkers

Venous blood was collected into gel serum tubes and centrifuged, and serum was stored at −80 °C until analysis. IL-4, IL-13, CXCL13, BDNF, NGF, YKL-40/CHI3L1, TREM-2, MCP-1, SNAP-25, neurogranin and pNF-H were measured by commercial human ELISA kits per manufacturer instructions, with optical density read at 450 nm and concentrations derived from standard curves. The IL-1β kit did not yield a reliable standard curve and was excluded from all analyses.

Venous blood was collected into gel serum tubes and centrifuged, and the serum was stored at −80 °C until analysis. IL-4, IL-13, C-X-C motif chemokine ligand 13 (CXCL13), brain-derived neurotrophic factor (BDNF), nerve growth factor (NGF), chitinase-3-like protein-1 (YKL-40/CHI3L1), triggering receptor expressed on myeloid cells 2 (TREM-2), monocyte chemoattractant protein-1 (MCP-1), synaptosomal-associated protein 25 (SNAP-25), neurogranin, and pNF-H were measured using commercial human enzyme-linked immunosorbent assay (ELISA) kits according to the manufacturers’ instructions. Optical density was measured at 450 nm, and concentrations were calculated from standard curves. The IL-1β kit did not yield a reliable standard curve; therefore, IL-1β was excluded from all analyses.

### 2.7. Statistical Analysis

Distributions were checked with the Shapiro–Wilk test and graphical inspection. Outliers were identified within-group per variable using a predefined Tukey IQR (1.5 × IQR) rule; flagged observations were excluded only from the relevant comparison (not deleted) and results were reported together with sensitivity analyses including all observations. Continuous variables were summarized as mean ± SD or median [IQR] and compared with independent-samples *t*-tests or Mann–Whitney U tests; categorical variables with chi-square/Fisher tests. Multiplicity was controlled with BH-FDR within predefined outcome families (biomarker panel; WISC-R; TAST/TODİL; sleep/psychosocial). Group differences in log-transformed biomarkers were additionally modeled with age- and sex-adjusted regression using HC3 robust standard errors, with the log-transformed concentration as the dependent variable, so that exp(B) − 1 expresses the percentage difference between groups. ROC analysis was exploratory. Within the asthma group, exploratory multiple regression related log-transformed, z-scored pNF-H, neurogranin and IL-13 (entered together, with age and sex) to z-scored language and cognitive outcomes, estimated with HC3 robust standard errors. Significance was set at *p* < 0.05.

## 3. Results

### 3.1. Sample Characteristics

The two groups were broadly comparable on demographic and background characteristics (Table 1). Median age was 9.83 years in the asthma group and 12.29 years in controls (*p* = 0.099), with no significant differences in sex, body mass index or parental education. Hearing was normal in all participants. Allergic rhinitis was present in 54.5% of the asthma group and in none of the controls, and 21 of 33 children with asthma were atopic; all had mild, well-controlled disease.

### 3.2. Serum Biomarkers

The clearest group difference was for pNF-H, higher in the asthma group (median 15.46 vs. 9.29; U = 712.0, *p* = 0.001, pFDR = 0.012). Neurogranin was also higher (153.10 vs. 120.30; U = 639.0, *p* = 0.012), but marginal after panel-level FDR (pFDR = 0.065). IL-13 tended to be higher but did not survive FDR (Table 2).

In age- and sex-adjusted models, the group difference persisted for pNF-H (B = 0.53; +70.3%, *p* = 0.002, pFDR = 0.016) and neurogranin (B = 0.64; +90.2%, *p* = 0.003, pFDR = 0.016), other analytes not differing (Table 3). Both differences were robust to outlier handling: in a sensitivity analysis retaining all observations, pNF-H and neurogranin remained significant after FDR (pNF-H *p* = 0.002, pFDR = 0.017; neurogranin *p* = 0.008, pFDR = 0.042).

### 3.3. Exploratory Group Discrimination (ROC)

**Figure 1:**
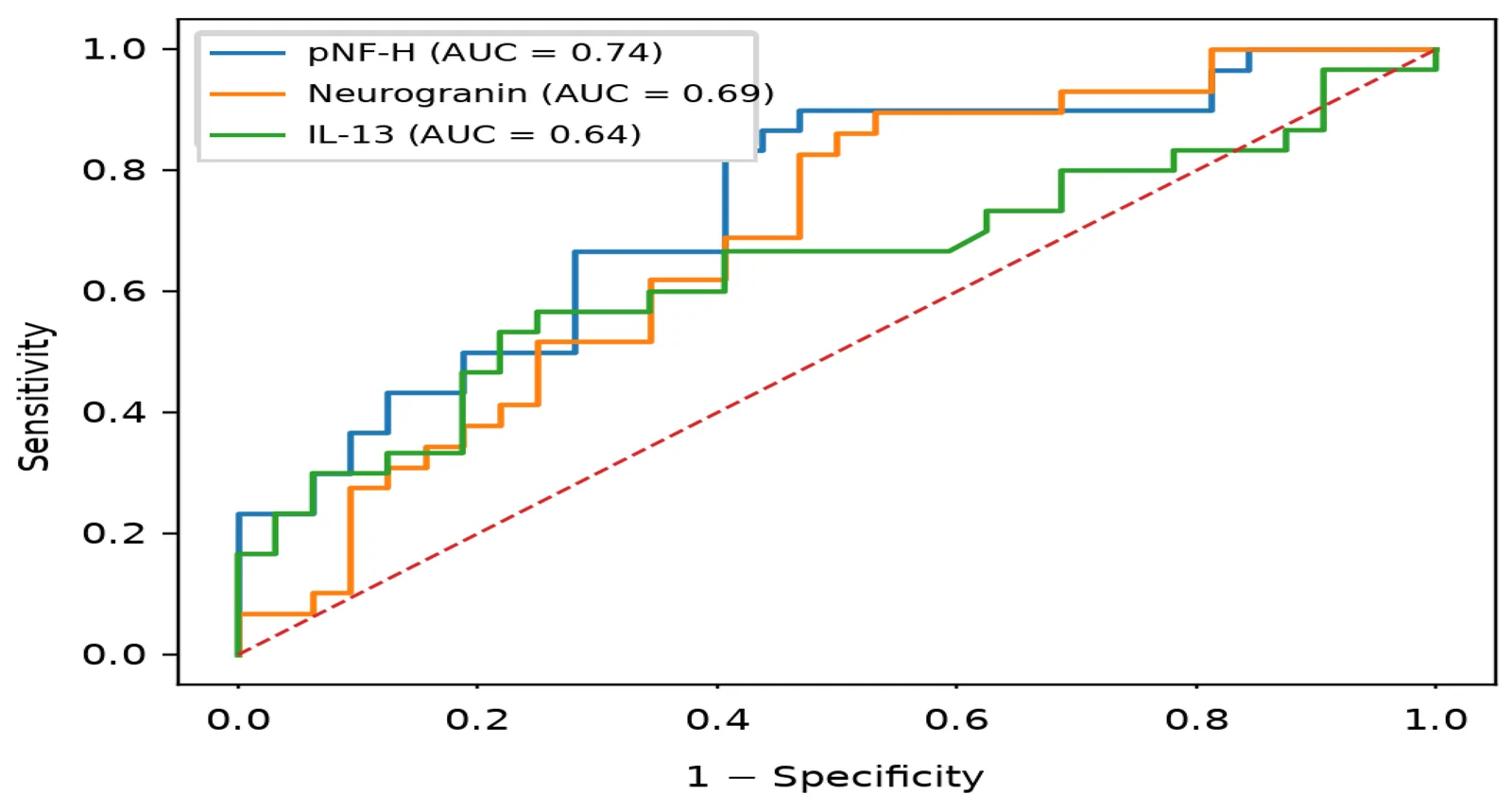
Exploratory receiver operating characteristic curves for pNF-H, neurogranin, and IL-13 for discrimination between children with asthma and healthy controls. The dashed diagonal line indicates chance-level discrimination.

In the ROC analysis, pNF-H yielded the highest AUC for discrimination between groups (AUC = 0.74; 95% CI: 0.61–0.86). At a cut-off of ≥9.32, sensitivity was 90%, specificity was 53%, and Youden’s J was 0.43. Neurogranin had an AUC of 0.69 (95% CI: 0.55–0.81); at a cut-off of ≥116.30, sensitivity was 90%, specificity was 47%, and Youden’s J was 0.37. IL-13 had an AUC of 0.64 (95% CI: 0.49–0.77); at a cut-off of ≥5.79, sensitivity was 57%, specificity was 75%, and Youden’s J was 0.32. These findings are exploratory because the cut-off values were derived and evaluated in the same sample. The absence of external validation limits conclusions regarding their diagnostic utility. The ROC analysis results for pNF-H, neurogranin, and IL-13 are summarized in Table 4, and the corresponding ROC curves are shown in Figure 1.

### 3.4. Cognitive, Language, Sleep and Psychosocial Measures

The principal cognitive outcome, WISC-R full-scale IQ, was lower in the asthma group (median 95.5 vs. 103.0; *p* = 0.008, pFDR = 0.024, r = 0.33), as were the verbal, verbal-comprehension and freedom-from-distractibility indices; performance IQ and perceptual organization did not differ (Table 5). Standardized language testing was consistent but more modest and is treated as supportive: TODİL listening was lower after FDR correction (pFDR = 0.014), whereas TAST total (pFDR = 0.056) and TODİL oral language (pFDR = 0.063) differed only nominally and did not survive correction. Sleep and psychosocial scales did not differ between groups.

### 3.5. Within-Asthma Biomarker Associations with Language and Cognition

To relate biomarkers to the language–cognitive phenotype directly within the clinical group, regression models were built in the asthma group only (without the group term), entering pNF-H, neurogranin and IL-13 together with age and sex. Neurogranin was the most consistent independent marker, positively associated with TODİL organizing, WISC-R vocabulary and WISC-R picture completion; IL-13 showed an independent negative association with picture completion (Table 6). At the model level, the biomarker block added significant variance for picture completion and vocabulary, but not for TODİL organizing or sleep (Table 7). These associations are exploratory and should be interpreted with multiplicity in mind.

## 4. Discussion

In this cross-sectional study of clinically well-controlled children with asthma, we observed selective differences in cognitive and language performance together with higher serum concentrations of pNF-H and neurogranin, against a background of comparable demographic, audiological, sleep and psychosocial characteristics. Because the groups were similar on these potential confounders, and because children with neurodevelopmental, psychiatric or other chronic conditions were excluded and hearing was screened audiometrically, the observed differences are less readily explained by behavioral, sleep or auditory factors. Given the cross-sectional design, however, they should be read as associations rather than causal effects.

The cognitive differences were selective rather than global, concentrated in verbal, verbal-comprehension and attention/working-memory-related indices, with no difference in performance IQ or perceptual organization broadly consistent with reports that childhood asthma is associated with difficulties in attention, processing speed, executive function, language and memory rather than a uniform reduction in general intelligence [8,9,10].

Standardized language testing was consistent with the cognitive pattern but more modest, and we treat it as supportive rather than a primary claim: TODİL listening was lower after FDR correction, whereas TAST total and TODİL oral language differed only nominally and did not survive correction, and no age-adjusted group analysis of these measures was available. The within-asthma associations described below, which were adjusted for age and sex, provide the stronger link between the biomarkers and language–cognitive performance.

The most robust biological finding was a higher serum concentration of pNF-H, which persisted after adjustment, was robust to outlier handling, and showed the highest, though moderate, group-level discrimination (AUC = 0.74). pNF-H is a phosphorylated neurofilament heavy-chain protein used as a marker of neuroaxonal integrity and injury [22,23]; measured peripherally and at the group level, we interpret it cautiously as a group-level signal potentially reflecting subtle differences in neuroaxonal turnover associated with chronic systemic inflammation, rather than as evidence of overt neuronal damage.

Neurogranin, a postsynaptic protein central to synaptic plasticity, was also higher; this difference was marginal after panel correction but significant after adjustment and is best described as a peripheral biomarker related to synaptic processes rather than as evidence of synaptic injury. Within the asthma group, neurogranin was positively associated with WISC-R vocabulary and picture completion and with TODİL organizing, and the biomarker block added meaningful variance for vocabulary and picture completion; IL-13 showed a single negative association with picture completion, which, given that IL-13 did not differ at the group level after correction, warrants caution. Notably, the direction for neurogranin (higher levels with better performance) is opposite to that reported in adult neurodegeneration, where higher neurogranin accompanies synaptic loss and decline [22,25]; in a developmental sample this may carry a different meaning, possibly related to plasticity or compensation.

The emphasis on pNF-H and neurogranin reflects their comparatively stronger findings in the present dataset and their complementary biological roles in neuroaxonal integrity and synaptic function. Nevertheless, neither marker is specific to asthma or neuroinflammation, and serum concentrations cannot be assumed to directly represent central nervous system concentrations or pathology. Their observed associations should therefore be regarded as preliminary and require confirmation using longitudinal designs and, where feasible, complementary neuroimaging or neurophysiological measures.

It is notable that the clearest group differences involved synaptic- and axonal-related markers (neurogranin, pNF-H) rather than classical type-2 inflammatory markers, and that most analytes (BDNF, NGF, MCP-1, CXCL13, TREM-2, SNAP-25, YKL-40) did not differ, suggesting that, in well-controlled asthma, not all neuroimmune components are equally engaged. Several plausible pathways could nonetheless link asthma to central nervous system function: chronic low-grade systemic inflammation and circulating cytokines [5], intermittent hypoxia and fragmented sleep [17,19], and processes suggested by adult cerebrospinal-fluid and imaging studies [20,21]; the mechanisms underlying the present associations cannot, however, be established from cross-sectional data.

Although the difference in age between the groups was not statistically significant, the asthma group was numerically younger than the control group. Given the age sensitivity of several cognitive and language measures, this imbalance may have influenced the observed group differences. Adjustment for age and sex did not materially alter the principal biomarker and cognitive findings; nevertheless, residual confounding cannot be excluded, particularly for language outcomes that were not evaluated in fully adjusted models. In addition, allergic rhinitis was substantially more prevalent in the asthma group. Because allergic rhinitis may adversely affect sleep quality, attention, and school performance, some of the observed cognitive and language differences may reflect the combined influence of asthma and allergic comorbidity rather than asthma alone. Therefore, the findings are most appropriately interpreted as characterizing a population of children with well-controlled asthma in whom allergic rhinitis was common.

Several limitations should be noted. The cross-sectional design precludes causal or directional inference, and peripheral blood biomarker concentrations may not directly reflect central nervous system processes. Although the between-group age difference was not statistically significant, the asthma group was numerically younger than the controls. Age-standardized WISC-R scores and age-based TODİL normative levels reduce, but do not eliminate, potential age-related confounding, particularly because the TAST total-correct score was not age-standardized and not all language outcomes were evaluated in fully adjusted models. Between-group differences in sex distribution may also have influenced the findings, and residual confounding by sex cannot be excluded in analyses that did not adjust for this variable. Allergic rhinitis was present in 54.5% of the asthma group and absent among controls; its close association with group membership prevented reliable separation of its independent contribution from that of asthma. Thus, the observed cognitive and language differences should not be attributed exclusively to asthma. The relatively small, single-center sample limits statistical power and generalizability, and the within-group biomarker–cognition models should be considered exploratory. Moreover, the inclusion of only children with mild, well-controlled asthma limits applicability to those with moderate-to-severe or poorly controlled disease. Larger, multicenter, longitudinal studies with appropriately matched groups are needed to confirm these preliminary findings.

Against these limitations, the study combined cognitive and standardized language assessment with sleep, psychosocial and audiological evaluation and a multi-analyte serum panel in the same, carefully characterized children; it screened and excluded major confounders including hearing loss and neuropsychiatric conditions; and it addressed multiplicity through predefined outcome families with FDR correction, confirming the leading biomarker differences in adjusted, outlier-robust models. Taken together, the findings suggest that the assessment of children with asthma may benefit from attention to specific verbal and attention domains and to standardized language measures rather than to global measures alone. They do not support routine screening or referral based on these markers; the appropriate next step is confirmation in larger, longitudinal and adequately powered studies incorporating neuroimaging, electrophysiology and more comprehensive neuropsychological assessment, and evaluating disease severity, duration and allergic comorbidity as modifiers.

## 5. Conclusions

Even in children with well-controlled asthma, differences in selected cognitive and language domains, particularly the WISC-R full-scale IQ, were observed compared with healthy peers. These differences were accompanied by higher circulating levels of biomarkers related to neuroaxonal and synaptic processes. Among these, pNF-H showed the most robust group-level difference, whereas neurogranin was additionally associated with specific language and cognitive measures within the asthma group. These findings should be regarded as preliminary and hypothesis-generating rather than as evidence supporting clinical diagnosis or screening. Confirmation in larger, longitudinal, and adequately powered studies is needed to clarify the potential interplay between neuroimmune processes, language development, and cognitive function in childhood asthma.

## Figures and Tables

**Table 1:** Demographic, clinical and audiological characteristics.

Variable	Asthma, Median [IQR]/*n* (%)	Control, Median [IQR]/n (%)	Statistic	*p*	ES
***Demographic***					
**Age (years)**	9.83 [7.75–13.50]	12.29 [10.88–12.67]	U = 429.0	0.099	0.20
**Sex (M/F)**	20/13	15/19	χ^2^ = 1.82	0.177	–
**BMI (kg/m^2^)**	21.46 [17.71–23.15]	18.71 [15.97–21.61]	U = 498.0	0.158	0.19
**Education year/grade**	4.00 [3.00–6.00]	5.00 [4.00–6.00]	U = 438.5	0.116	0.19
**Maternal education ≥ high school**	17 (51.5)	20 (58.8)	Fisher	0.627	0.07
**Paternal education ≥ high school**	19 (57.6)	22 (64.7)	Fisher	0.621	0.07
***Asthma-related clinical features***					
**Age at onset (years)**	4.00 [2.00–6.00]	–	–	–	–
**Duration (years)**	5.50 [3.00–8.00]	–	–	–	–
**Allergic rhinitis**	18 (54.5)	0 (0.0)	Fisher	<0.001	0.59
**Atopic/non-atopic asthma**	21/12	–	–	–	–
**FEV1% predicted**	91.0 [84.0–98.0]	–	–	–	–
***Audiological findings***					
**Bilateral PTA (dB HL)**	8.33 [5.00–10.00]	7.50 [5.00–10.00]	U = 518.0	0.578	0.07
**Bilateral normal hearing**	33 (100.0)	34 (100.0)	Fisher	1.000	0.00

*Note*: Continuous variables median [IQR], categorical *n* (%); Mann–Whitney U/chi-square–Fisher. ES = r (continuous) or φ (categorical). Asthma-specific variables were not compared with controls.

**Table 2:** Serum biomarker levels by group (after outlier exclusion).

Biomarker	Asthma, Median [IQR]	Control, Median [IQR]	U	*p*	pFDR	r
**BDNF**	1.137 [0.867–1.429]	1.094 [0.868–1.471]	490.5	0.978	0.978	0.00
**CXCL13**	165.60 [124.90–228.80]	169.35 [132.05–229.73]	455.0	0.902	0.978	0.02
**YKL-40/CHI3L1**	27.46 [21.68–35.50]	25.52 [20.73–29.06]	492.0	0.534	0.797	0.08
**IL-4**	96.33 [72.73–122.10]	82.10 [62.38–119.70]	528.0	0.489	0.797	0.09
**IL-13**	6.028 [3.742–9.276]	4.146 [3.466–5.428]	611.5	0.065	0.238	0.24
**MCP-1**	170.80 [147.10–211.40]	158.85 [141.78–223.25]	472.0	0.580	0.797	0.07
**Neurogranin**	153.10 [130.30–195.20]	120.30 [92.25–154.00]	639.0	0.012	0.065	0.32
**NGF**	114.20 [95.54–151.80]	116.80 [87.90–157.05]	406.5	0.410	0.797	0.11
**pNF-H**	15.46 [11.25–20.72]	9.29 [6.76–14.70]	712.0	0.001	0.012	0.42
**SNAP-25**	2.595 [2.381–3.419]	2.806 [2.381–4.605]	426.0	0.359	0.797	0.12
**TREM-2**	4.431 [3.823–5.118]	4.563 [3.646–4.985]	477.5	0.851	0.978	0.02

*Note*: Concentrations in pg/mL, median [IQR]. Outliers identified within-group by the Tukey IQR rule; pFDR = BH-adjusted *p*; r = rank-biserial effect size.

**Table 3:** Age- and sex-adjusted regression for log-transformed biomarkers.

Biomarker	B	Difference, % (95% CI)	*p*	pFDR	R^2^
**BDNF**	0.12	12.9 (−14.2 to 48.4)	0.386	0.607	0.03
**CXCL13**	0.01	1.2 (−19.3 to 27.0)	0.916	0.916	0.01
**YKL-40/CHI3L1**	0.01	1.0 (−15.1 to 20.2)	0.908	0.916	0.01
**IL-4**	0.18	19.3 (−8.1 to 54.9)	0.184	0.398	0.05
**IL-13**	0.21	23.9 (−3.8 to 59.6)	0.098	0.358	0.12
**MCP-1**	0.06	6.2 (−11.7 to 27.6)	0.525	0.641	0.04
**Neurogranin**	0.64	90.2 (24.6 to 190.6)	0.003	0.016	0.21
**NGF**	−0.24	−21.7 (−45.4 to 12.2)	0.183	0.398	0.07
**pNF-H**	0.53	70.3 (21.9 to 137.9)	0.002	0.016	0.20
**SNAP-25**	−0.12	−11.3 (−26.6 to 7.3)	0.217	0.398	0.03
**TREM-2**	0.06	6.1 (−10.1 to 25.3)	0.484	0.641	0.04

*Note*: Group coded asthma = 1, control = 0; adjusted for age and sex with HC3 robust standard errors; dependent variable = log concentration, so percentage difference = exp(B) − 1.

**Table 4:** ROC analysis of selected biomarkers.

Biomarker	*n*	AUC	95% CI	Cut-Off Value	Sensitivity	Specificity	Youden’s J
pNF-H	67	0.74	0.61–0.86	≥9.32	90%	53%	0.43
Neurogranin	67	0.69	0.55–0.81	≥116.30	90%	47%	0.37
IL-13	67	0.64	0.49–0.77	≥5.79	57%	75%	0.32

Note: AUC = area under the curve; CI = confidence interval. Cut-off values were determined using Youden’s index.

**Table 5:** Cognitive, language, sleep and psychosocial measures by group.

Variable	Asthma	Control	U/χ^2^	*p*	pFDR	ES
***WISC-R (median [IQR])***						
**Verbal IQ**	92.0 [84.0–100.0]	101.5 [94.0–109.0]	U = 694.0	0.014	0.027	0.30
**Performance IQ**	97.0 [88.0–104.0]	102.5 [94.0–111.0]	U = 660.5	0.061	0.073	0.23
**Full-scale IQ**	95.5 [85.2–100.8]	103.0 [94.5–110.5]	U = 707.5	0.008	0.024	0.33
**Verbal Comprehension**	93.0 [86.0–101.0]	102.0 [94.0–110.0]	U = 689.5	0.018	0.027	0.29
**Perceptual Organization**	96.0 [88.0–105.0]	101.0 [93.0–109.0]	U = 638.0	0.118	0.118	0.19
**Freedom from Distractibility**	90.0 [82.0–97.0]	99.0 [90.0–106.0]	U = 701.0	0.006	0.024	0.34
***Language (TAST/TODİL)***						
**TAST total correct**	25.0 [22.0–28.0]	28.0 [25.0–30.0]	U = 684.0	0.016	0.056	0.29
**TODİL—Listening (level)**	Average	Average	U = 704.0	0.002	0.014	0.29
**TODİL—Organizing (level)**	Average	Average	U = 513.5	0.616	0.616	0.06
**TODİL—Speaking (level)**	Average	Average	U = 646.5	0.101	0.141	0.16
**TODİL—Grammar (level)**	Average	Average	U = 625.0	0.232	0.271	0.13
**TODİL—Semantics (level)**	Average	Average	U = 634.0	0.084	0.141	0.17
**TODİL—Oral Language (level)**	Average	Average	U = 672.0	0.027	0.063	0.24
***Sleep (SDSC)***						
**Sleep disturbance total**	44.0 [38.0–48.0]	40.5 [35.0–48.0]	U = 472.0	0.475	0.554	0.09
**Sleep disturbance risk, n (%)**	11 (33.3)	8 (23.5)	χ^2^ = 0.78	0.377	0.528	0.11
***Psychosocial***						
**CBCL Total Competence**	38.0 [34.0–43.0]	42.0 [36.0–47.0]	U = 645.5	0.097	0.427	0.20
**CBCL Total Problem**	54.0 [48.0–61.0]	50.0 [44.0–57.0]	U = 637.0	0.122	0.427	0.19
**Anxiety—Total**	31.0 [22.0–40.0]	27.0 [19.0–36.0]	U = 612.0	0.286	0.528	0.13

*Note*: Median [IQR] or *n* (%); Mann–Whitney U/chi-square–Fisher; pFDR = within-family BH-adjusted p; ES = r or φ. TODİL entries are normative levels (both within the Average band); U/p reflect the ordinal comparison.

**Table 6:** Significant independent biomarker coefficients in within-asthma multiple robust regression.

Dependent Variable	Biomarker	*n*	β	95% CI	*p*	ΔR^2^
**TODİL organizing**	Neurogranin	33	0.79	0.09 to 1.50	0.028	0.23
**WISC-R picture completion**	Neurogranin	33	0.81	0.21 to 1.40	0.008	0.25
**WISC-R picture completion**	IL-13	33	−0.49	−0.95 to −0.03	0.039	0.25
**WISC-R vocabulary**	Neurogranin	33	0.64	0.16 to 1.12	0.008	0.15

*Note*: β = standardized coefficient; ΔR^2^ = variance added when pNF-H, neurogranin and IL-13 are added to the age- and sex-adjusted base model. Biomarkers log-transformed and z-scored; dependent variables z-scored; HC3 robust standard errors. **WISC-R,** Wechsler Intelligence Scale for Children–Revised.

**Table 7:** Incremental contribution of the biomarker block.

Dependent Variable	*n*	ΔR^2^	Adj. R^2^	Block *p*
**WISC-R picture completion**	33	0.25	0.35	0.026
**WISC-R vocabulary**	33	0.15	0.35	0.035
**TODİL organizing**	33	0.23	0.32	0.118
**Sleep total**	33	0.05	−0.14	0.715

*Note*: Block *p* is a Wald test of the pNF-H, neurogranin and IL-13 coefficients jointly; ΔR^2^ is variance added to the age- and sex-adjusted base model.

## Data Availability

The data presented in this study are available from the corresponding author upon reasonable request.
